# Study on the Relationship between Nano-Morphology Parameters and Properties of Bitumen during the Ageing Process

**DOI:** 10.3390/ma13061472

**Published:** 2020-03-24

**Authors:** Mengyuan Zhang, Xuancang Wang, Wengang Zhang, Longting Ding

**Affiliations:** 1 School of Highway, Chang’an University, Xi’an 710064, China; zmyy2018@163.com (M.Z.); dltphd2018@163.com (L.D.); 2 School of Civil and Architectural Engineering, Shandong University of Technology, Zibo 255000, China; ziwuzizwg@sdut.edu.cn

**Keywords:** atomic force microscopy, nano-morphology parameters, viscosity of bitumen, bee-like structure, ageing

## Abstract

Thermo-oxidative ageing is one of the main factors affecting bitumen performance. At present, the research on bitumen ageing has entered the micro stage. The purpose of this paper was to study the relationship between nano-morphology parameters and properties of bitumen of bitumen during the ageing process. To this end, bitumen with different ageing degrees was prepared in this paper, and Atomic force microscopy samples with different cooling rates were prepared. The relationship between ageing degree of bitumen and nano-morphology parameters was analyzed. A functional relationship model between nano-morphology parameters and properties of bitumen was established. The results show that the percentage of bee-like structure area ($P_{\mathrm{bee}-\mathrm{like}}$), maximum amplitude ($H_{\max}$) and roughness ($R_{q}$) increased with the increase of ageing degree. the percentage of bee-like structure area, the maximum amplitude and the roughness increase with the increase of cooling rate. With the increase of the percentage of bee-like structure area, the maximum amplitude and the roughness, the viscosity of bitumen at 60 °C increases, penetration decreases, and softening point increases. There is a nonlinear relationship between the nano-morphology parameters and properties of bitumen.

## 1. Introduction

The ageing of bitumen is the result of components changes [1]. Light, heat, oxygen, water and other factors can cause the ageing of bitumen, which accounts for most of the total ageing [2,3,4]. In recent years, research on bitumen ageing has mainly focused on the ageing mechanism [5,6,7,8], the performance prediction during ageing [9,10], and the development of anti-ageing agent [11,12,13]. There are many ways to obtain aged bitumen, such as by using Rolling Thin Film Oven and Thin Film Oven. Xu et al. [13] used molecular dynamics to study the effect of thermal-oxidative ageing on bitumen properties, including the nano-aggregation behaviour of asphaltene molecules and the translation ability of asphalt molecules. Infrared spectroscopy is also a common method to measure the carbonyl absorption peak of bitumen before and after ageing to characterize the ageing degree [14,15,16,17,18,19]. In addition, a dynamic shear rheological test (DSR) [20,21,22,23] and gel permeation chromatography (GPC) [24,25] can also be used to study the ageing behaviour and ageing mechanism of bitumen. However, these methods can not reveal the relationship between bitumen micro-properties and ageing properties.

With the development of testing technology, the research on bitumen ageing has gradually turned to micro and nano-scale. In recent years, fluorescence microscopy has been widely used in the microscopic study of polymer modified bitumen [26,27,28]. The fluorescence microscope uses ultraviolet light as a light source to illuminate the modified bitumen to fluoresce the polymer, thereby observing the microstructure of the modified bitumen under a microscope [29,30]. However, fluorescence microscopy is powerless for the micro-morphology of matrix bitumen [31,32]. In addition, atomic force microscopy (AFM) is becoming more and more popular among professionals in the field of bitumen micro-detection [33,34,35]. Atomic force microscopy was invented in 1985 by Gerd Binning, who was working in Zurich Research Center of International Business Machines Corporation [36,37,38]. The purpose of AFM is to enable non-conductors to be observed by scanning probe microscopy (SPM). The biggest difference between atomic force microscopy (AFM) and scanning tunneling microscope (STM) is that it does not use an electron tunneling effect, but detects atomic contact, atomic bonding, van der waals force or casimir effect to present the surface properties of samples [39]. The principle of atomic force microscopy is as follows [40,41,42,43]. Fix one end of the microcantilever that is sensitive to weak forces, and a tiny tip is on the other end. During the test, the tip of the needle is in light contact with the surface of the sample. There is a very weak repulsion force between the tip atoms and the surface atoms of the sample, keep this repulsion force constant, then the microcantilever with the tip will undulate in a direction perpendicular to the surface of the sample. Using optical detection or tunneling current detection, the position of the microcantilever corresponding to each point of the scanning can be measured so that the surface topography information can be obtained.

Atomic force microscopy (AFM) has been widely used in many fields due to the high resolution (nanometer level) and less requirement for samples. In recent years, the application of AFM in the field of bitumen has provided a new method for studying the morphological characteristics of bitumen at the nanoscopic perspective. The application of AFM in bitumen field can be traced back to 1996. Loeber [44] observed the existence of bee-like structure in bitumen by using AFM. The cause of bee-like structure is inconclusive now, but most scholars believe that the existence of asphaltene and wax is the cause of a bee-like structure. A lot of research work has been carried out on the nanostructure of bitumen based on AFM, which mainly focus on the size of the bee-like structure, percentage of bee-like structure area, roughness etc., but there are still two problems, which have not been solved yet: (1) The calculation method of bitumen nano-morphology parameters is not uniform. (2) Lack of quantitative relationship between nano-morphological parameters and macro-technical indicators of bitumen.”

In this paper, AFM was used to study the ageing properties of bitumen. The calculation method of nano-morphology parameters of bitumen AFM is standardized, and the quantitative relationship model between nano-morphology parameters and macro-technical indexes of bitumen is established. Bitumen with different ageing degree was prepared, and Atomic force microscopy (AFM) samples with different cooling rates were prepared respectively. The relationship between the ageing degree of bitumen and nano-morphology parameters was analyzed. A functional relationship model between nano-morphology parameters and properties of bitumen was established. For a long time, it has been difficult for researchers to establish a quantitative relationship between the nano-morphology parameters and the technical properties of bitumen. The research method of this paper provides a reference for improving the calculation accuracy of the bitumen nano-morphology parameters, and the research results provide a new idea for the study of the quantitative relationship between the technical properties and the nano-morphology parameters of bitumen, which is of great significance.

## 2. Test Design

### 2.1. Materials

The bitumen (A-90) used in this paper was produced by Zhonghai Asphalt Co., Ltd. (Binzhou, China). The technical indicators of the bitumen used in this paper are shown in Table 1. It should be noted that viscosity is measured by the Vacuum Decompression Capillary Method, the penetration measurement temperature is 25 °C, the softening point is measured by the Ring and Ball Method. The measurement methods of the indicators in Table 1 are the relevant methods specified in “Standard Test Methods of Bitumen and Bituminous Mixtures for Highway Engineering (JTG E20-2011)” [45].

### 2.2. Test Design

**Figure 1:**
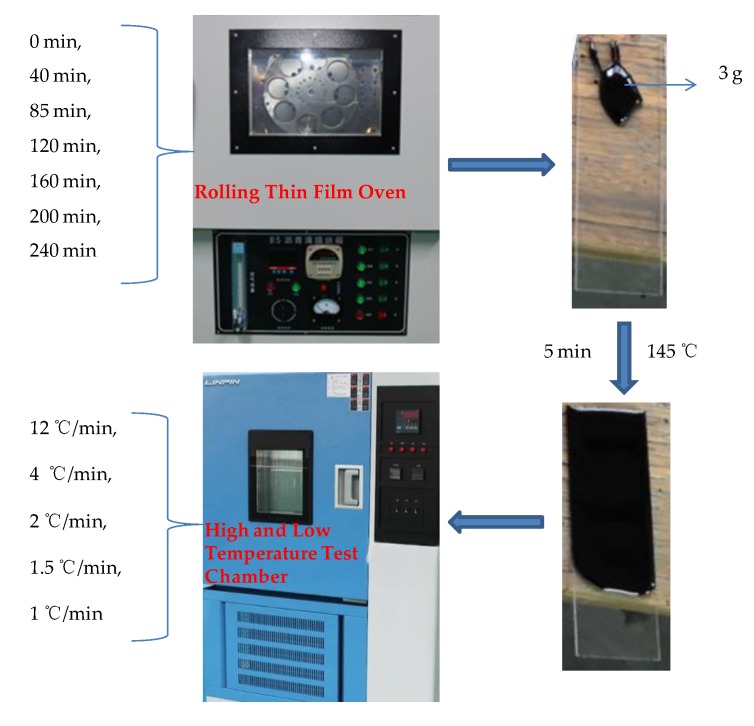
The preparation process of the atomic force microscopy (AFM) sample.

**Figure 2:**
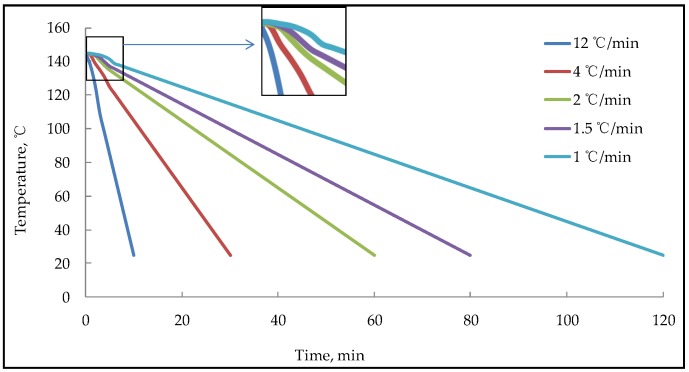
The measured cooling rate of the high and low temperature test chamber.

The rolling thin film oven (85#) used in this paper was produced by Cangzhou Hengsheng Weiye Highway Instrument Co., Ltd. (Cangzhou, China). The high and low temperature test chamber was produced by Linping Instrument Co., Ltd. (Shanghai, China), its cooling rate can reach 15 °C/min at the fastest. Rolling thin film oven test (RTFOT) was used to obtain bitumen with the different ageing degree. The ageing time is 0 min, 40 min, 85 min, 120 min, 160 min, 200 min and 240 min, respectively. An amount of 3 g of the aged bitumen was dropped on a glass slide and kept in the high and low temperature test chamber at 145 °C for 5 min, which made the bitumen form a thin layer with similar thickness and smooth surface. Then turn on the cooling mode and cooled to 25 °C at a cooling rate of 12 °C/min, 4 °C/min, 2 °C/min, 1.5 °C/min and 1 °C/min, respectively. Then the nano-morphology parameters of bitumen with the different ageing degree and cooling rate was tested by AFM at 25 °C. The preparation process of the AFM sample is shown in Figure 1. The measured cooling rate of the high and low temperature test chamber is shown in Figure 2.

As can be seen from Figure 2, the cooling rate is not linear within 0 to 5 min, which shows that the cooling rate is small at first, then gradually faster, and finally, reaches the expected rate. This phenomenon is caused by the residual temperature in the high and low temperature chamber. Since the bitumen temperature in the unstable stage of the cooling rate is always above 135 °C, the bitumen is still in a liquid state, so the instability phenomenon in the initial stage of cooling is neglected in the study of this paper.

### 2.3. The Nano-Morphology Parameters

**Figure 3:**
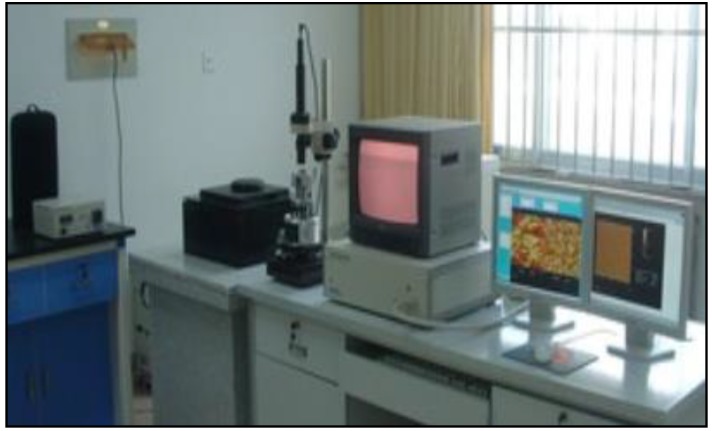
AFM used in this paper.

In this paper, the nanostructure of bitumen was observed by using AFM. The AFM was produced by Veeco Company (New York, the United States of America). The horizontal resolution is 0.2 nm, the scanning range is 10 μm × 10 μm, the test temperature is 25 °C, and the number of samples are 512. The AFM used in this paper is shown in Figure 3.

**Figure 4:**
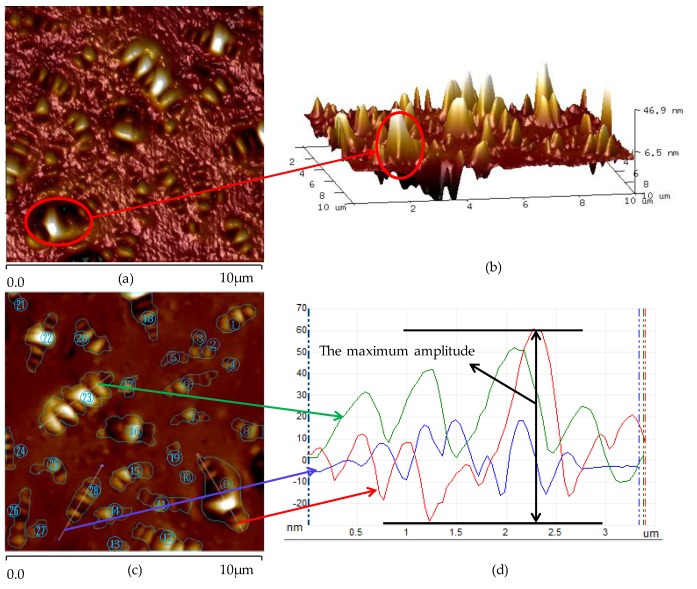
Bitumen AFM images. (**a**) two-dimensional height images; (**b**) three-dimensional height images; (**c**) bee-like structure analysis images; (**d**) maximum amplitude.

The nano-morphology parameters used in this paper are introduced in conjunction with Figure 4.

It can be seen from Figure 4 that there are many bee-like structures in bitumen AFM images, which are composed of undulating peaks and troughs in three-dimensional images. In the two-dimensional image, the percentage of bee-like structure area to the total area of the image is the percentage of bee-like structure area ($P_{\mathrm{bee}-\mathrm{like}}$). In the three-dimensional image, the maximum height difference between the peak and trough of the bee-like structure is the maximum amplitude ($H_{\max}$). The overall degree of three-dimensional fluctuation of bitumen AFM is roughness ($R_{q}$).

There are many bee-like structures in the AFM image. In order to calculate the area of a bee-like structure, each bee-like structure is numbered, from 1 to i, as shown in Figure 4c. Then the area of each bee-like structure is read by Image-Pro-Plus software [46,47]. $P_{\mathrm{bee}-\mathrm{like}}$ is the sum of each bee-like structure area divided by the total area of the image. As shown in Equation (1).

(1) $$P_{\mathrm{bee}-\mathrm{like}}=\frac{\sum_{1}^{i}A_{i}}{A}\times 100\%$$

where $A_{i}$ is the area of the bee-like structure numbered i and A is the total area of the AFM image.

As shown in Figure 4c,d, the height fluctuation curve is obtained along with the bee-like structure, and the maximum and the minimum height of the curve can be read. $H_{\max}$ can be calculated according to Equation (2).

(2) $$H_{\max}=h_{\max}-h_{\min}$$

where $h_{\max},\;h_{\min}$ are the maximum and the minimum height in the AFM image respectively.

$R_{q}$ can be calculated according to Equation (3).

(3) $$R_{q}={\left(\frac{\iint {\left[h\left(x,\;y\right)-h_{0}\right]}^{2}\mathrm{dS}}{\iint \mathrm{dS}}\right)}^{1/2}$$

where S is the scanning area, 10 μm × 10 μm in this paper. h(x, y) is a height function, nm. $h_{0}$ is the reference height, which can be calculated according to Equation (4), nm.

(4) $$h_{0}=\frac{\iint h\left(x,\;y\right)\mathrm{dS}}{\iint \mathrm{dS}}$$

## 3. Results and Discussion

### 3.1. Effect of Ageing Time and Cooling Rate on Nano-Morphology of Bitumen

**Figure 5:**
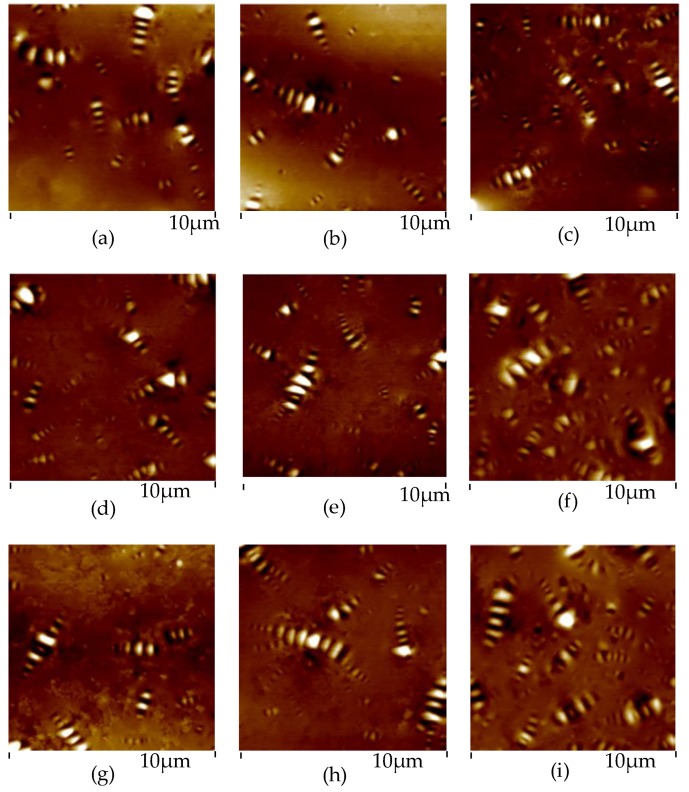
AFM images of bitumen with different ageing degree. The cooling rate of (**a**–**c**) is 12 °C/min; The cooling rate of (**d**–**f**) is 2 °C/min; The cooling rate of (**g**–**i**) is 1 °C/min; The ageing time of (**a**,**d**,**g**) is 0 min; The ageing time of (**b**,**e**,**h**) is 85 min; The ageing time of (**c**,**f**,**i**) is 240 min.

**Figure 6:**
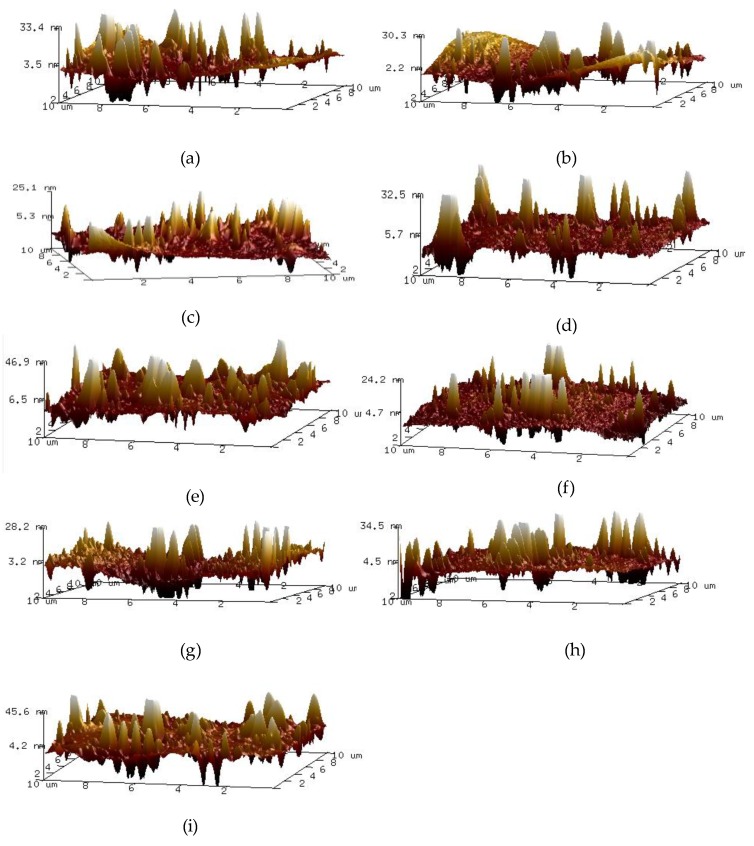
The three-dimensional nanoscopic images of bitumen with different ageing degree. The cooling rate of (**a**–**c**) is 12 °C/min; The cooling rate of (**d**–**f**) is 2 °C/min; The cooling rate of (**g**–**i**) is 1 °C/min; The ageing time of (**a**,**d**,**g**) is 0 min; The ageing time of (**b**,**e**,**h**) is 85 min; The ageing time of (**c**,**f**,**i**) is 240 min.

In this paper, the nano-morphology of A-90 bitumen with different ageing degree and cooling rate was tested. The results are shown in Figure 5 and Figure 6.

**Figure 7:**
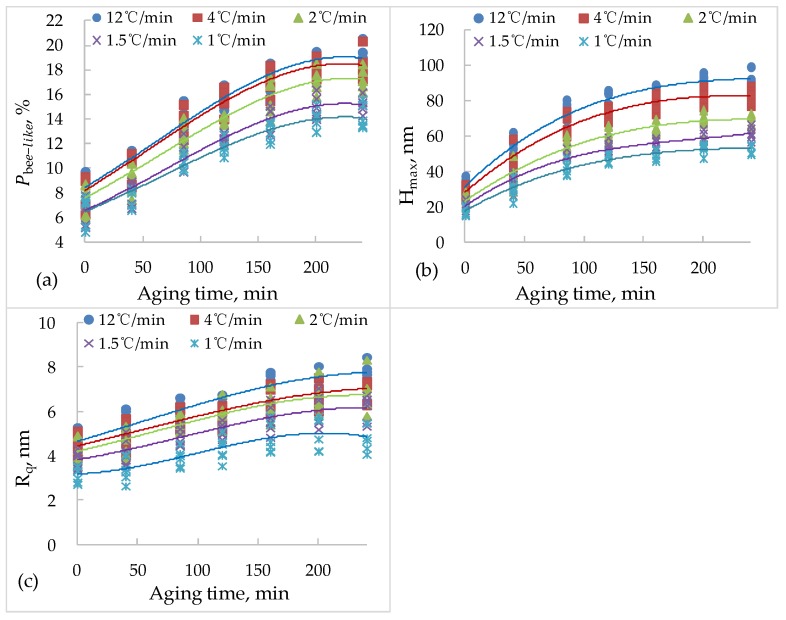
The nano-morphology parameters of bitumen with different ageing time and cooling rate. (**a**) $P_{\mathrm{bee}-\mathrm{like}}$; (**b**) $H_{\max}$; (**c**) $R_{q}$.

As can be seen from Figure 5 and Figure 6 that as the ageing degree of bitumen increases, the nano-morphology in AFM images also changes. The longer the ageing time, the larger the volume of bee-like structure, the higher the fluctuation degree, and the larger the percentage of bee-like structure area. The nano-morphology of bitumen under different cooling rates is also different. As the cooling rate increases, the bee-like structure is small and dense, but the degree of undulation is increased. Figure 7 shows the nano-morphology parameters of bitumen with different ageing times and cooling rates.

According to the method in Section 2, the ageing time in this paper is 0 min, 40 min, 85 min, 120 min, 160 min, 200 min and 240 min, respectively. The cooling rate of 12 °C/min, 4 °C/min, 2 °C/min, 1.5 °C/min and 1 °C/min, respectively, the target temperature is 25 °C. As shown in Figure 7, $P_{\mathrm{bee}-\mathrm{like}}$, $H_{\max}$ and $R_{q}$ increase with the increase of ageing degree. For the $P_{\mathrm{bee}-\mathrm{like}}$, no matter the cooling rate, the average area of the bee-like structure is 2.2 times of that of the non ageing bitumen, the $H_{\max}$ is 2.9 times of that of the non aging bitumen, and the $R_{q}$ is 2.6 times of that of the non ageing bitumen. This shows that ageing has a great influence on the s nano-morphology parameters of bitumen. The reason is that with the ageing process, the light components in bitumen decrease gradually, which leads to increases in the asphaltene proportion. The existence of asphaltene is the main cause of the bee-like structure formation, so the ageing process actually promotes the development of the bee-like structure. Figure 7 also shows that $P_{\mathrm{bee}-\mathrm{like}}$, $H_{\max}$ and $R_{q}$ will increase while the cooling rate increases. Under the same ageing time, the asphalt with a faster cooling rate has greater $P_{\mathrm{bee}-\mathrm{like}}$, $H_{\max}$ and $R_{q}$.

### 3.2. Relationship between the Nano-Morphology Parameters and the Bitumen Properties

**Figure 8:**
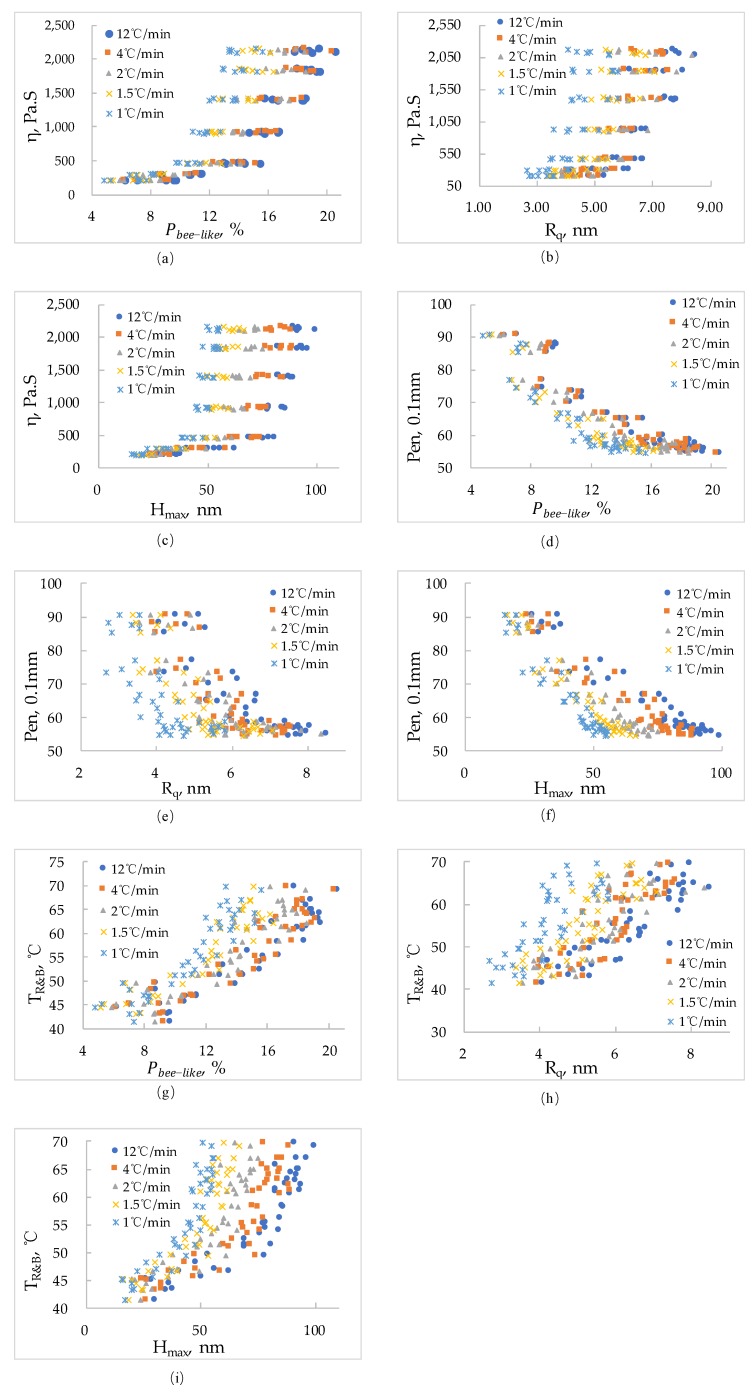
Relationship between asphalt nano-parameters and technical indexes. (**a**) η and $P_{\mathrm{bee}-\mathrm{like}}$; (**b**) η and${\;R}_{q}$; (**c**) η and $H_{\max}$; (**d**) Pen and $P_{\mathrm{bee}-\mathrm{like}}$; (**e**) Pen and${\;R}_{q}$; (**f**) Pen and $H_{\max}$; (**g**) T_R&B_ and $P_{\mathrm{bee}-\mathrm{like}}$; (**h**) T_R&B_ and${\;R}_{q}$; (**i**) T_R&B_ and $H_{\max}$.

The index of bitumen viscosity, such as viscosity at 60 °C, penetration and softening point was tested in this paper. The test results are shown in Figure 8.

Figure 4 shows that with the increase of $P_{\mathrm{bee}-\mathrm{like}}$, the viscosity of bitumen at 60 °C increases, the penetration decreases and the softening point increases. With the increase of $R_{q}$, the viscosity at 60 °C increases, the penetration decreases and softening point increases; with the increase of $H_{\max}$, the viscosity at 60 °C increases, the penetration decreases and the softening point increases. At the same time, the cooling rate has a certain effect on the results. It can be seen from the test data that there is a non-linear relationship between each nano-parameter and the bitumen technical properties. Based on the numerical analysis of the test data, a relationship model between nano-morphology parameters and technical properties of bitumen is proposed. As shown in Equation (5).

(5) $$\mathrm{TI}=a\times {\left(b\times {P_{\mathrm{Nano}}}^{3}+c\times {P_{\mathrm{Nano}}}^{2}+d\times P_{\mathrm{Nano}}+e\right)}^{T^{\prime }}+f\times T^{\prime }+g$$

where TI is the technical indexes; $P_{\mathrm{Nano}}$ is the nano-morphology parameters; $T^{\prime }$ is the cooling rate, °C/min; a, b, c, d, e, f and g are the regression coefficients.

According to Equation (5), the test results in Figure 4 are fitted. The fitting results are shown in Table 2.

**Figure 9:**
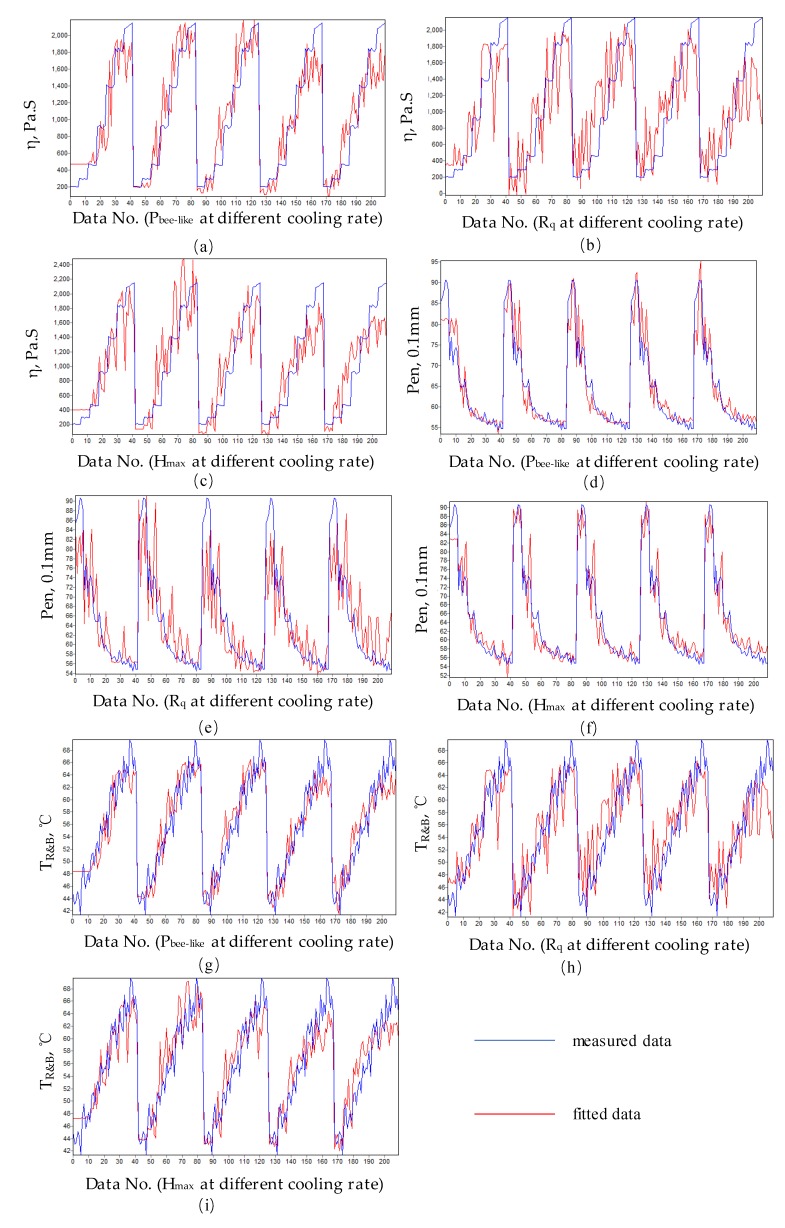
Fitting effect. (**a**) Equation (6); (**b**) Equation (7); (**c**) Equation (8); (**d**) Equation (9); (**e**) Equation (10); (**f**) Equation (11); (**g**) Equation (12); (**h**) Equation (13); (**i**) Equation (14).

Figure 9 shows the fitting effect.

Table 2 and Figure 9 show that the relationship between the nano-morphology parameters of bitumen and the indexes characterizing bitumen viscosity can be quantified. From the fitting effect, the correlation coefficient can reach about 90%, which shows that the correlation between them is significant. This makes the study of the nano-morphology parameters of bitumen has a certain significance. This method also provides a reference for studying the relationship between the nanoscopic morphology parameters and the macroscopic technical indexes of bitumen.

## 4. Conclusions

In this paper, bitumen with different ageing degrees was prepared, and AFM samples with different cooling rates were prepared respectively. The relationship between ageing degree of bitumen and nano-morphology parameters was analyzed. A functional relationship model between nano-morphology parameters and properties of bitumen was established. The research method of this paper provides a reference for improving the calculation accuracy of the bitumen nano-morphology parameters and the research results provide a new idea for the study of the quantitative relationship between the technical properties and the nano-morphology parameters of bitumen, which is of great significance. The main conclusions are as follows:

(1) The percentage of bee-like structure area, the maximum amplitude and the roughness will increase while the ageing degree of bitumen increases.
(2) In the preparation process of AFM samples, the faster the cooling rate, the smaller the average area of the bee-like structure, but the larger the percentage of the bee-like structure area and the maximum amplitude and roughness.
(3) While the percentage of the bee-like structure area, the maximum amplitude and roughness increase, the viscosity at 60 °C will increase, the penetration will decrease and the softening point will increase.
(4) The relationship between the nano-morphology parameters of bitumen and the indexes of bitumen viscosity accords to $\mathrm{TI}=a\times {\left(b\times {P_{\mathrm{Nano}}}^{3}+c\times {P_{\mathrm{Nano}}}^{2}+d\times P_{\mathrm{Nano}}+e\right)}^{T^{\prime }}+f\times T^{\prime }+g$.

## Figures and Tables

**Table 1:** Technical indicators of A-90 bitumen.

Test Items		Value
Penetration, 25 °C, 0.1 mm		88.1
Ductility, 15 °C, cm		101.9
Viscosity at 60 °C, Pa·s		202.411
Softening point, °C		43.7
RTFOT ^1^	Penetration ratio, %	71.9
	Residual ductility, 15 °C, cm	33.7
	Mass loss, %	0.3

^1^ RTFOT, Rolling Thin Film Oven Test.

**Table 2:** A-90 bitumen technical indexes and nano-morphology parameters relationship fitting equations.

No.	TI	$P_{\mathrm{Nano}}$	a	b	c	d	e	f	g	R^2^, %
Equation (6)	η	$P_{\mathrm{bee}-\mathrm{like}}$	2266.6	−0.0006	0.02	−0.18	0.39	35.1	51.94	95.04
Equation (7)	η	$R_{q}$	2455.2	−0.006	0.07	−0.08	−0.44	52.8	−289.7	88.26
Equation (8)	η	$H_{\max}$	3359.3	−0.00003	0.0004	−0.01	0.06	27.5	61.4	92.23
Equation (9)	Pen	$P_{\mathrm{bee}-\mathrm{like}}$	−38.83	0.0004	−0.02	0.43	−1.6	−1.1	94.24	96.83
Equation (10)	Pen	$R_{q}$	−63.70	0.003	−0.08	0.63	−0.79	−2.4	115.12	89.13
Equation (11)	Pen	$H_{\max}$	−43.14	0.00002	−0.0001	0.05	−0.53	−1.1	95.15	97.85
Equation (12)	$T_{R\&B}$	$P_{\mathrm{bee}-\mathrm{like}}$	25.62	−0.0003	0.01	0.02	−0.28	0.6	41.77	98.26
Equation (13)	$T_{R\&B}$	$R_{q}$	32.23	−0.002	−0.002	0.3	−0.57	0.9	−35.9	89.31
Equation (14)	$T_{R\&B}$	$H_{\max}$	33.83	−0.00001	0.001	0.01	−0.21	0.4	42.03	93.1

η is the viscosity at 60 °C; Pen is the penetration; T_R&B_ is the softening point.
